# Isolation and detection of circulating tumour cells from metastatic melanoma patients using a slanted spiral microfluidic device

**DOI:** 10.18632/oncotarget.18641

**Published:** 2017-06-27

**Authors:** Carlos A. Aya-Bonilla, Gabriela Marsavela, James B. Freeman, Chris Lomma, Markus H. Frank, Muhammad A. Khattak, Tarek M. Meniawy, Michael Millward, Majid E. Warkiani, Elin S. Gray, Mel Ziman

**Affiliations:** ^1^ School of Medical and Health Sciences, Edith Cowan University, Perth, Western Australia, Australia; ^2^ Department of Health, Perth, Western Australia, Australia; ^3^ Transplantation Research Program, Boston Children’s Hospital and Department of Dermatology, Brigham and Women’s Hospital, Harvard Medical School, Boston, Massachusetts, USA; ^4^ Harvard Stem Cell Institute, Harvard University, Cambridge, Massachusetts, USA; ^5^ Department of Medical Oncology, Fiona Stanley Hospital, Murdoch, Western Australia, Australia; ^6^ School of Medicine and Pharmacology, University of Western Australia, Crawley, Western Australia, Australia; ^7^ Department of Medical Oncology, Sir Charles Gairdner Hospital, Nedlands, Western Australia, Australia; ^8^ School of Mechanical and Manufacturing Engineering, Australian Center for NanoMedicine, University of New South Wales, Sydney, New South Wales, Australia; ^9^ School of Pathology and Laboratory Medicine, University of Western Australia, Crawley, Western Australia, Australia

**Keywords:** circulating tumour cells (CTCs), metastatic melanoma, slanted spiral microfluidics

## Abstract

Circulating Tumour Cells (CTCs) are promising cancer biomarkers. Several methods have been developed to isolate CTCs from blood samples. However, the isolation of melanoma CTCs is very challenging as a result of their extraordinary heterogeneity, which has hindered their biological and clinical study. Thus, methods that isolate CTCs based on their physical properties, rather than surface marker expression, such as microfluidic devices, are greatly needed in melanoma. Here, we assessed the ability of the slanted spiral microfluidic device to isolate melanoma CTCs via label-free enrichment. We demonstrated that this device yields recovery rates of spiked melanoma cells of over 80% and 55%, after one or two rounds of enrichment, respectively. Concurrently, a two to three log reduction of white blood cells was achieved with one or two rounds of enrichment, respectively. We characterised the isolated CTCs using multimarker flow cytometry, immunocytochemistry and gene expression. The results demonstrated that CTCs from metastatic melanoma patients were highly heterogeneous and commonly expressed stem-like markers such as PAX3 and ABCB5. The implementation of the slanted microfluidic device for melanoma CTC isolation enables further understanding of the biology of melanoma metastasis for biomarker development and to inform future treatment approaches.

## INTRODUCTION

Metastatic disease occurs in approximately 10% of melanoma patients at diagnosis and drastically reduces survival in the first year after diagnosis [1, 2]. Despite remarkable advances in the treatment of melanoma, only 20% of patients with metastatic spread show long lasting responses (>2 years) to current treatments [3–6]. Thus, a better understanding of the pathogenic mechanisms underlying metastatic spread will ultimately lead to the discovery of biomarkers that predict, with high reliability, clinical outcome and treatment response, a critical unmet need for melanoma.

Circulating tumour cells (CTCs), are the tumour seeds responsible for metastatic haematogenous dissemination of solid tumours and arise from both primary and secondary tumours. Since CTCs can be detected and isolated from blood, phenotypic and genetic characterisation of these cells provides insightful information about the biology of melanoma metastasis, as well as the evolving phenotype and genotype of all concurrent tumours within a patient [7]. The ease of access of CTCs from blood for repeated sampling, exemplifies CTCs as invaluable biomarkers for real-time monitoring of clinical status and treatment response [8, 9]. In melanoma, cumulative evidence supports a role for CTCs in the prediction of clinical outcome and monitoring of therapeutic response [10–14].

Despite the potential biomarker utility of CTCs, the isolation and detection of these cells is a significant technical challenge that must be overcome to achieve a better understanding of their biology and role in metastasis, as well as for their implementation in the clinic. Firstly, CTCs are extremely rare in the blood of melanoma patients (i.e. 1-10 cells per 8 mL of blood) [10–12, 15–17], demanding methods that isolate CTCs at a high recovery rate and with a low background of white blood cells (WBCs). Secondly, the high phenotypic heterogeneity observed in melanoma CTCs adds significant complexity, with no single marker detecting the majority of melanoma CTCs [10, 13, 15, 16, 18]. This is in contrast with the use of the cell surface marker, EpCAM (epithelial cell adhesion molecule) [19] and the internal marker, cytokeratin, standard CTC markers for epithelial cancer cells [20–23]. Thus, conventional isolation methods that depend on the expression of cell surface markers (i.e. label-dependent methods) are not optimal in melanoma and provide a biased isolation outcome [7]. Instead, label-independent isolation methods (i.e. microfluidic devices) may provide a solution, as they utilise differential cell size, density and rigidity to enrich CTCs from blood [18, 24–27]. Various microfluidic devices have been developed and validated for isolation of CTCs from patients with different types of cancer, mainly of epithelial origin, such as micro-fabricated filters [28, 29], electric-based approaches [30], the herringbone CTC-Chip [31], CTC-iChip [32], Cluster-Chip [18], Parsortix [26, 33] and spiral-based chips [24, 34].

In melanoma, microfluidic devices (i.e. herringbone CTC-Chip, CTC-iChip or Cluster-Chip) have been used to isolate single or cluster CTCs from metastatic patients before [13, 18, 32] and during treatment with MAPK inhibitors [13]. However, additional isolation methods that yield a viable, label-free and more diverse fraction of CTCs are urgently required for the isolation of melanoma CTCs. The implementation of such methods in melanoma is also critical for downstream applications, such as genetic and phenotypic characterisation of CTCs, cell culture and CTC-derived xenotransplantion, which will provide an insight into the biology of CTCs and melanoma metastasis [18, 35–38].

Spiral microfluidic devices, which rely on “Dean drag” forces, have been recently developed to isolate CTCs based on cell size, deformability and density with high purity, viability and recovery efficiency [24, 34, 39]. These spiral devices have demonstrated their superiority over the only FDA-approved platform for CTC enumeration, CellSearch® (Janssen Diagnostics LLC), in the recovery of CTCs in breast and lung cancer patients [34]. Particularly, the slanted spiral device has been shown to yield recovery rates of over 80% with spiked breast and bladder cancer cells, and to isolate and detect CTCs in almost all the analysed blood samples from patients with metastatic breast and lung cancer [24]. But no report has been shown its utility for the isolation of melanoma CTCs. This device has the advantageous capability of being able to process 8 mL of blood sample in less than 10 minutes to yield CTCs at relatively high purity (500 WBCs per mL of processed blood) [24], compared to over an hour by other microfluidic devices [18, 25, 31–33, 39]. In addition, short term cell cultures of CTCs from breast cancer patients have been established after isolation of CTCs through this slanted device, demonstrating the high viability of CTCs isolated through this device, as a result of the low shear stress caused to the cells [38].

Here, we validate the ability of the slanted spiral microfluidic device to isolate a viable, label-free and heterogeneous population of CTCs from the blood of melanoma patients with metastatic disease. Furthermore, this enrichment allowed us to interrogate the phenotype and gene expression profile of CTCs isolated from metastatic melanoma patients prior to treatment, using multimarker flow cytometry, gene expression analysis and immunofluorescence staining.

## RESULTS

### Recovery and purity of melanoma cells with different cell size

**Figure 1:**
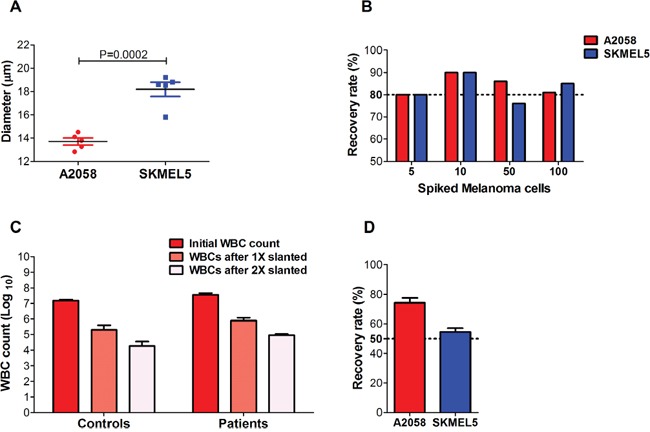
Recovery rates and purity of melanoma cells isolated using a slanted spiral microfluidic device Melanoma cells were spiked into blood from healthy donors. **(A)** Comparison of the mean diameters of melanoma cell lines A2058 and SKMEL5, after five consecutive passages, yielded a statistically significant difference between mean diameters of both cell lines (t-test, P=0.0002). **(B)** Recovery rates of 5, 10, 50 and 100 CellTracker^™^ red-labelled A2058 and SKMEL5 cells spiked in blood from healthy donors after 1X slanted enrichment (n=3). No statistically significant differences were found in the recovery rates for A2058 (P=0.376) and SKMEL5 cell lines (P=0.102) between different spiked amounts. **(C)** WBC counts in the blood of healthy donors (controls, n=3) and metastatic melanoma patients (n=5) before and after the first and second round of enrichment with the slanted microfluidic device. A one-way ANOVA analysis determined a highly significant depletion of WBCs after the second round of enrichment with the slanted device in both controls (P<0.0001) and patients (P<0.0001). **(D)** Recovery rates of 50 A2058 and SKMEL5 cells spiked into blood after two rounds of slanted enrichment (controls, n=4). Error bars represent one standard deviation from the mean values across replicates.

To determine the efficiency of the slanted spiral microfluidic device to isolate circulating melanoma cells of different cell sizes, we firstly classified the melanoma cell lines 1205Lu, A2058, SKMEL5, UACC62, WM164 and WM793, based on their cell size, inferred by forward scatter values in a flow cytometer platform (Supplementary Figure 2). A2058 and SKMEL5 were respectively identified as the cell lines with the smallest (mean cell diameter: 14 μm) and largest cell size (mean cell diameter: 18 μm), and the mean cell diameters significantly differed between these two melanoma cell lines (P=0.0002) (Figure 1A). We spiked blood from healthy individuals with increasing amounts of these cell lines and processed them through the slanted spiral microfluidic device. An overall average recovery rate of 83% was observed across all the spiked blood samples with either A2058 or SKMEL5 cells, and no significant differences were observed in the recovery rates for each melanoma cell line, regardless of the number of cells spiked (Figure 1B). To assess the purity of the enriched fraction, we quantified WBCs in the sample before and after processing through the slanted device. We achieved a 2 log reduction in the number of WBCs after processing the sample once (1X slanted) through this device (mean: 2.0 × 10^5^; range: 0.7 - 4.2 × 10^5^) compared to the initial WBC count (mean: 1.5 × 10^7^, range: 1.2 - 1.7 × 10^7^) (Figure 1C).

A second slanted enrichment was performed (2X slanted) with the purpose of further reducing the WBC background for future genetic interrogation of the CTC fraction. This resulted in a total 3 log WBC reduction (mean: 1 × 10^4^; range: 0.7-4 × 10^4^) after 2X slanted enrichment (Figure 1C). We measured the recovery rates of the 2X slanted enrichment by spiking 50 A2058 or SKMEL5 cells into healthy donor blood to determine whether a second slanted enrichment could yield an enriched sample with a higher purity without loss in recovery of melanoma cells. We obtained an average recovery rate of 74% for A2058 and 55% for SKMEL5 cells (Figure 1D).

Next we proceeded to analyse CTCs in melanoma patient blood samples, firstly assessing the purity and secondly identifying CTCs in the CTC-enriched samples after slanted enrichment. To determine the level of purity of CTC fractions from patients after enrichment with the slanted spiral device, we counted WBCs before and after processing 8 mL of blood from 5 metastatic melanoma patients once and twice through the slanted spiral device. On average, we again found a 2-3 log reduction in WBCs relative to the initial WBC count (mean: 3.5 × 10^7^ WBCs (range: 2.4 × 10^7^ - 4.9 × 10^7^), after 1X slanted, 7.7 × 10^5^ WBCs (range: 245,000 – 465,500) and after 2X slanted, 8.6 × 10^4^ WBCs (range: 49,105 – 123,203). Thus, a second round of enrichment through the slanted device demonstrated its ability to significantly deplete WBCs with high significance in both control and patient samples (P<0.0001), even though WBC levels were higher in patients than in healthy controls (Figure 1C). Samples from these 5 patients were included in the CTC detection experiments detailed below.

### Detection of CTCs by flow cytometry before and after slanted enrichment

In order to demonstrate the presence of melanoma cells in the ‘CTC fraction’ after enrichment through the slanted spiral device, we processed blood samples from 10 metastatic melanoma patients, prior to treatment (baseline) (Table 1). The ‘CTC fraction’ was stained with multiple markers and analysed by flow cytometry, as previously described by Gray et al. [10]. Results were compared to the number of CTCs observed in a separate sample from each patient without slanted enrichment but analysed by flow cytometry. This flow cytometry assay identifies subpopulations of CTCs based on their positivity for melanoma-associated markers, MCAM (melanoma cell adhesion molecule) and MCSP (melanoma-associated chondroitin sulphate proteoglycan), and/or their positivity for melanoma-initiating cell markers, ABCB5 (ATP-binding cassette subfamily B member 5), CD271 and RANK (Receptor Activator of NFkβ pathway) [10].

**Table 1:** Demographic and clinical information of metastatic melanoma patients

Characteristic		n	% of total
**Total patients enrolled**		**20**	
**Age at enrolment (years)**			
Median	66		
Range	34 - 80		
**Gender**			
Male		13	65%
Female		7	35%
**Stage of disease at baseline**			
**Stage IV**			
M1a		4	20%
M1b		1	5%
M1c^α^		15	75%
**Mutational status of tumour**			
***BRAF***			
V600E		11	55%
V600R		1	5%
V600K		1	5%
Other BRAF*		1	5%
Wildtype BRAF^+^		6	30%
**Prior treatment for (Baseline):**			
Pembrolizumab		10	50%
Dabrafenib + Trametinib		10	50%

* Case with BRAF N581S, NRAS G12D, MET E355K mutations. ^+^ Case with wild type status for BRAF and NRAS, but with KIT L576P and CTNNB1 S37F mutations. ^α^ Three cases with brain metastases.

**Figure 2:**
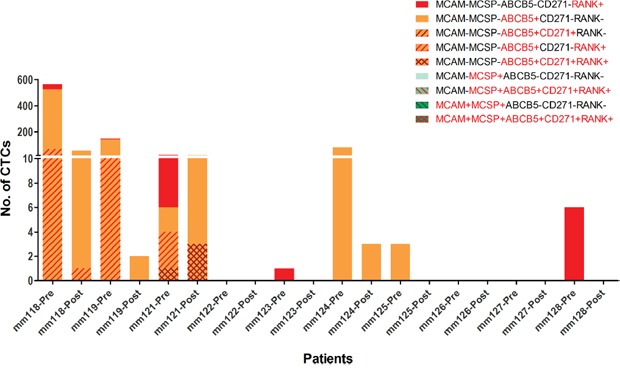
Diversity of melanoma CTCs pre and post slanted enrichment Comparison of phenotype and number of CTCs identified before and after slanted enrichment in 10 metastatic melanoma patients using multimarker flow cytometry. Bars indicate the number of CTCs detected by flow cytometry in each individual, with each phenotype indicated by a colour and pattern.

Using this approach, we detected CTCs in 7 out of 10 patients, with a median of 5 CTCs (range: 1 - 565) in 8 mL of blood, before any slanted enrichment. As previously described [10], we observed high diversity in the phenotype of CTCs detected in these patients before slanted enrichment. ABCB5 was the most common marker of CTCs, and RANK was the second most common marker, expressed either alone or in combination with ABCB5. Melanoma-associated markers MCSP and MCAM were not detected on any CTCs isolated from the 10 metastatic melanoma patients (Figure 2). We also detected CTCs post slanted enrichment in 4 out the 7 CTC positive patients, though at lower numbers (1-62 CTCs) (Figure 2). The 6 cases with no CTCs detected after processing through the microfluidic device had low or no CTC counts (0-6 CTCs) prior to any enrichment. In contrast, cases with detected CTCs after slanted processing had much higher initial CTC counts (24-565 CTCs in 8 mL of blood). The phenotype of the cells isolated by the slanted device were present prior to enrichment, albeit at different frequencies. The melanoma-initiating marker ABCB5 remained the most common marker after slanted enrichment and was observed to be more highly represented after enrichment than the other CTC markers.

### Molecular detection of melanoma-specific transcript levels in enriched fractions

We also characterised the melanoma cells in the ‘CTC fraction’ after slanted enrichment by assessing the gene expression of 5 melanoma-associated genes [40]. The genes included: *MLANA/MART1* (melanoma antigen recognized by T cells), *TYR* (tyrosinase), *MAGEA3* (melanoma antigen family A3), *PAX3* (paired box protein Pax-3 isoform 3) and *ABCB5.* These genes were selected based on their known exclusive expression in melanoma cells and their undetected expression by this RT-PCR assay in WBC samples from healthy individuals (n=5). Moreover, these genes are either known markers of melanoma pathology, due to their high expression in melanoma tumours, and/or involved in melanoma pathogenesis [41–44].

**Figure 3:**
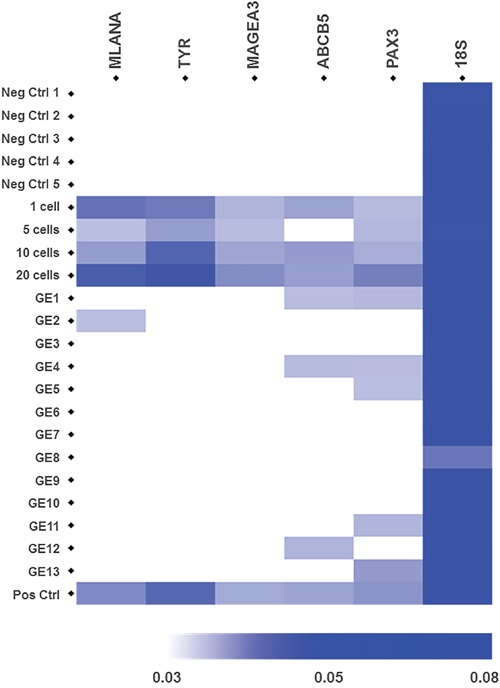
Gene expression of 5 melanoma-specific genes in samples from healthy controls, spiked samples and CTC fractions from metastatic melanoma patients after slanted enrichment Heatmap represents the expression levels of the melanoma-associated genes *MLANA, TYR, MAGEA, ABCB5* and *PAX3*, which were assessed by RT-PCR. The 18S rRNA gene was used as an endogenous control. Cellular fractions after slanted processing of the blood from 5 healthy controls were used as negative controls. Spiked samples with 1, 5, 10 and 20 A2058 melanoma cells in a background of 1 × 10^5^ WBCs were used to demonstrate the detection level of this assay. CTC-enriched fractions from 13 metastatic melanomas were interrogated. Spiked samples with 20 A2058 cells were used as positive controls in every run. Each heatmap square corresponds to the reciprocal value of the Ct values for each target gene (1/Ct) for a given sample.

First we assessed whether we can detect these 5 genes in RNA extracted from samples containing 1, 5, 10 and 20 melanoma cells spiked into 100,000 WBCs, the level of WBC background observed after 2X slanted enrichment (Figure 1C). Transcripts of *MLANA*, *TYR*, *MAGEA3*, *PAX3*, and *ABCB5* were successfully identified from a single melanoma cell, as reflected by the increase in reciprocal Ct values (1/Ct) (Figure 3).

In patient samples, we detected melanoma specific transcripts in 7 out of 13 (54%) CTC enriched fractions from metastatic melanoma patients. In particular, the *PAX3* transcripts were detected exclusively in 3 out of the 7 positive CTC-enriched samples, while also detected along with *ABCB5* in another 2 cases (thus 5/7 cases). *ABCB5* and *MLANA* transcripts were detected alone in one patient each (Figure 3). Differences in 18S rRNA Ct values across all analysed samples were a result of differential WBC counts in the CTC fractions across all patients. Interestingly, all the 7 patients with detected PAX3, ABCB5 or MLANA transcripts by RT-PCR had metastatic disease in distant organs (stage M1c) and two of three cases with brain metastases had CTC fractions positive by RT-PCR, for *MLANA* and *PAX3*. Furthermore, four of the patients with detected melanoma-specific transcripts had CTC counts higher than 155 CTCs per 8 mL of blood; whilst the remaining three had from 1 to 11 CTCs per 8 mL of blood, detected by flow cytometry before slanted enrichment. In contrast, the patients with undetected transcripts had at least 1 CTC per 8 mL of blood (n= 5, 1-81 CTCs) detected by flow cytometry before slanted enrichment, with only one case negative for CTCs.

### Detection of melanoma CTCs using immunostaining

**Figure 4:**
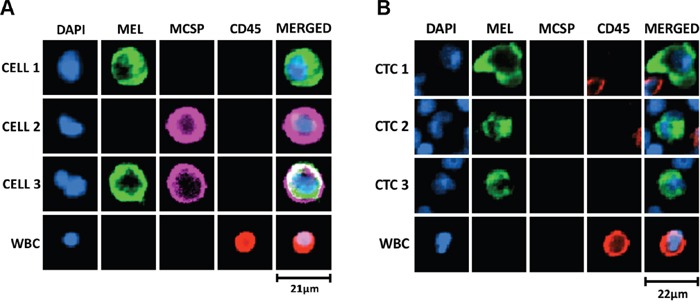
Melanoma-specific multimarker immunofluorescence staining for detection of melanoma CTCs **(A)** Optimisation of immunostaining protocol in WBC samples spiked with A2058 cells, cytospun onto glass slides, fixed, permeabilised and immunostained with a combination of cell surface, MCSP (Alexa Fluor® 647, purple), and intracellular -MEL- (gp100, S100 and MLANA; Alexa Fluor® 488, green) markers. DAPI was used for nuclei staining and CD45 (PE, red) as a leukocytic marker. Differential expression of MCSP and MEL markers was observed in A2058 cells. **(B)** Representative melanoma CTCs detected by this immunofluorescence staining in metastatic melanoma patient blood processed twice through the slanted spiral microfluidic device. Melanoma CTCs had an average cell size of 16 μm (range: 13 – 21 μm). MCSP staining was not detected in any of the isolated melanoma CTCs from patient samples. Scale bar represents size of the cell in μm.

To further demonstrate the presence of CTCs in the enriched fractions, we developed a melanoma-specific multimarker immunostaining protocol. Markers included MCSP, a melanoma cell surface specific marker, together with a mixture of internal melanocytic markers, gp100 (glycoprotein 100), S100, and MLANA, hereafter called “MEL” immunostaining. Firstly, we optimised this staining in A2058 cells spiked into WBCs from healthy volunteers (Figure 4A). Subsequently, we used this immunostaining protocol to detect melanoma CTCs in enriched fractions isolated from 7 metastatic melanoma patients using the slanted spiral device. Using this staining protocol, we observed CTCs in 3 cases (43%), with 1, 3 and 4 CTCs in 8 mL of blood. All detected CTCs were positive for the combination of intracellular markers “MEL” but negative for MCSP staining and exhibited an average cell size of 16 μm (range: 13 – 21 μm) (Figure 4B). Furthermore, two of the 3 cases with detected CTCs by immunostaining had melanoma metastasis in distant organs (stage M1c), and the remaining one had subcutaneous and lymph node metastases.

## DISCUSSION

The isolation of CTCs is critical for the study of the role of these cells in the metastatic process as well as their utility as biomarkers in the clinical management of cancer. Therefore, the implementation of methods that enable the isolation of viable and label-free CTCs is of high biological and clinical significance. This is particularly important for isolation of CTCs from melanoma patients due to their diversity, since label-based capture significantly biases the number of CTCs that can be identified [10, 15, 16]. Here, we report the successful isolation of CTCs from the blood of metastatic melanoma patients using a slanted spiral microfluidic device. We demonstrate, through downstream interrogation of the CTC phenotype by flow cytometry, immunostaining and gene expression analyses, that label-free enrichment of melanoma CTCs successfully isolates heterogeneous subpopulations of cells.

The slanted spiral microfluidic device enabled us to retrieve melanoma cells with different cell sizes with a recovery rate similar to that observed for breast and bladder cancer cell lines [24]. This demonstrates the reliability of this device to differentially enrich for cancer cells regardless of cell size and cancer type; a very important feature in further applications of this device. In addition, we achieved close to a 3 log reduction of WBCs after a second round of slanted enrichment without markedly affecting enrichment efficiency. Despite a reduction of 2-3 log fold of WBCs in healthy donors and patients after one or two rounds of slanted enrichment, a higher leukocytic background was observed than that previously reported at 5 × 10^3^ WBCs per 8 mL of processed sample after 1X slanted enrichment [24]. A higher leukocytic background in the CTC fraction in patient samples relative to controls is likely due to the observed leukocytosis exhibited by metastatic melanoma patients [45]. Further strategies, such as CD45 depletion [27] might better reduce the number of leukocytes after slanted enrichment for further molecular and genomic characterisation of melanoma CTCs. However, despite the need to further improve the leukocytic background yielded by the slanted device in controls (10,000 WBCs per 8 mL of blood) and patients (86,000 WBCs per 8 mL of blood), these values are better than those reported for CTC-iChip (32,000 WBCs per mL of control blood), which was previously used for isolation of melanoma CTCs [46]. More recent microfluidic platforms, such as Parsortix (Angle plc), have shown promising results in regards to WBC depletion, with approximately 2800 WBCs detected after processing 4 mL of blood from healthy volunteers [33]. Therefore, a study comparing the recovery, WBC depletion in patients and CTC isolation efficiencies, for melanoma, of the slanted spiral microfluidic relative to other microfluidic devices [18, 26, 32] is strongly encouraged. This approach will determine the best platform for melanoma CTC isolation to be utilised in further clinical studies. Nonetheless, the microfluidic platforms previously used for isolation of melanoma CTCs are not readily available [13, 18, 32].

Phenotypic analysis of CTCs before and after slanted enrichment, using multimarker flow cytometry [10], showed reduction in the number and diversity of CTCs being enriched by the slanted spiral microfluidic device. This is mainly caused by a preferential enrichment of CTCs expressing ABCB5 over the rest of the melanoma CTCs expressing other markers, particularly RANK expressing CTCs. Since the slanted spiral device isolates CTCs based on their differential cell size and density [24], it is possible that ABCB5+ cells vary in these physical characteristics and might be preferentially enriched. Ozkumur et al. [46] have indicated a variability of melanoma CTCs in cell size after microfluidic isolation, but it is still unclear whether CTC subtypes do in fact vary in cell size and density. The differential recovery rates observed for the melanoma cell lines A2058 and SKMEL5 after a second round of slanted might support this preferential enrichment. It is possible that these cell lines may not only differ in size but also in other physical properties such as cell density or deformability, explaining the differential recovery observed. Nevertheless, despite the reduction in number and diversity of melanoma CTCs after slanted enrichment, we did obtain a heterogeneous fraction of CTCs, identified by another two independent methods, melanoma-specific gene expression assays and immunofluorescent staining.

Our immunocytochemistry staining protocol identified CTCs expressing melanocytic markers in 43% of metastatic melanoma patients. However, this method would miss cells expressing melanoma-initiating markers. The evidence that a vast majority of melanoma CTCs express “stem cell” markers such as ABCB5, shown by flow cytometry and *PAX3*, by gene expression, argues for inclusion of such markers to increase the CTC detection rate by immunocytochemistry.

The most significant finding from this study is the detection of the expression of melanoma-specific genes in CTC fractions after slanted enrichment. We found melanoma-associated gene expression in 54% of analysed CTC fractions. All patients with CTCs positive by RT-PCR for *PAX3*, *ABCB5* or *MLANA* transcripts exhibited metastatic disease in distant organs (M1c) prior to treatment and two of the three cases with brain metastases were positive for *PAX3* or *MLANA*. Interestingly, the fact that *PAX3* and *ABCB5* were detected in 6 out 7 cases with metastatic disease, positive by RT-PCR, might provide further evidence supporting the role of these genes in melanoma progression [14, 44, 47–50], but more importantly in the biology of melanoma CTCs. Thus, while this study was not aimed at determining a correlation between the expression of these melanoma-specific genes and clinical indicators, such as tumour burden or location, our findings warrant confirmation in a larger cohort.

Interestingly, *PAX3* transcripts were detected in five out of the seven CTC positive cases, being co-expressed with *ABCB5* in two of those. The specific role of *PAX3* in the biology of melanoma CTCs requires further investigation. PAX3 is a transcription factor that directs melanocytic differentiation from neural crest cells, and is involved in stem cell maintenance and cell migration of melanoblasts [51–54]. *PAX3* is also highly expressed in primary and secondary melanoma tumours [47, 49]. It is possible that PAX3 might orchestrate melanoma metastasis by maintenance of the “stem cell” phenotype of these cells during migration [47, 51, 53, 54]. In addition, *PAX3* has been described as a mediator of a drug-tolerance phase prior to the development of acquired resistance to targeted MAPK inhibition, via upregulation of *MITF* [55]. Thus, the detection of *PAX3* in CTC fractions might also have clinical implications. In this study, *PAX3* transcripts were detected in two out of ten patients prior to MAPK treatment, and three out of ten pembrolizumab patients. Further study of the relationship between *PAX3* expression in melanoma CTCs with early response to MAPK inhibitors, in a larger cohort of patients, may provide an insight into its role in the development of acquired resistance and its biomarker utility.

ABCB5, a tumour initiating or “stem cell” marker known to be involved in tumour resistance to chemotherapy and targeted therapy in melanoma, identifies a subset of slow-cycling tumour cells with increased potential to initiate metastases [44, 50, 56]. Previously, we reported [14] the expression of *ABCB5* in unenriched whole blood from melanoma patients by RT-PCR and showed that *ABCB5* transcripts were present in 40% of melanoma cases at all stages, particularly in those with disease recurrence (49%) and metastatic disease (52%). Here we found *ABCB5* gene expression detected by RT-PCR in 23% of all CTC-enriched patient samples with no detection of *ABCB5* transcripts in ‘CTC fractions’ from healthy donors. ABCB5 was also found to be the most commonly expressed marker by melanoma CTCs detected by flow cytometry in unenriched and enriched samples. These findings highlight the importance of ABCB5 in CTC biology and the intrinsic heterogeneous nature of melanoma CTCs. Thus, further molecular and functional studies aiming to investigate the role of ABCB5 in melanoma CTCs, and interrogate CTC phenotypes during disease evolution as well as throughout treatment will unveil the relationship between ABCB5 and melanoma progression.

The phenotypic and molecular heterogeneity of melanoma CTCs, reported in this study, indicate that the real heterogeneity of melanoma CTCs may still be underestimated. Previous studies have detected melanoma CTCs in 40% [11], 57% [16], 52% [10], 79% [13] of patients, using CellSearch® (Janssen Diagnostics LLC), isolation by size of epithelial tumour cells (ISET) platform, multimarker flow cytometry and the herringbone CTC-Chip, respectively. These rates are similar to the CTC detection rates we observed of 54% (RT-PCR) and 43% (immunostaining) in this study. However, it is likely that differences in the CTC detection rates across these studies may be attributed to the extraordinary heterogeneity of melanoma CTCs both within and between patients [10]. Based on previous studies [10, 15, 16], we expect that not all melanoma CTCs express the same markers. Therefore, it is possible that the current markers used in downstream approaches for CTC detection may constrain our ability to detect the vast majority of melanoma CTC types. Thus, studies aimed at the capture and study of the whole range of melanoma CTCs to identify the real diversity of these cells are urgently needed in order to pinpoint better markers that minimise false negative results.

Challenges in the isolation and detection of CTCs, particularly in melanoma, have hindered studies aiming at unveiling the molecular mechanisms underlying melanoma CTC biology relative to that of bulk tumour cell biology. Nevertheless, although we did not analyse patient-matched tumour samples in this study, we have previously compared melanoma cell subpopulations between patient matched blood and metastatic tumours [10]. This revealed that cell populations expressing melanoma initiating markers, such as ABCB5 and RANK, are more common amongst CTCs than in the matching tumour, suggesting a preferential selection for certain tumour cell subtypes in the blood. Thus, further investigation of CTC heterogeneity relative to tumour diversity will provide a much clearer understanding of the biology of CTCs and the metastatic process in melanoma. The isolation of viable melanoma CTCs may allow future biological characterisation through CTC-derived cell cultures and xenotransplantation [18, 35–38]. Furthermore, research is required to validate the biomarker utility of viable CTCs, as indicated previously by label-dependent methods [10–12, 16].

Ultimately, the isolation of viable, label-free heterogeneous populations of melanoma CTCs using this slanted enrichment device will facilitate future studies aimed at unveiling the molecular mechanisms and biology of melanoma CTCs and their role in melanoma metastasis and response to therapy.

## MATERIALS AND METHODS

### Cell culture of melanoma cell lines

The melanoma cell lines 1205Lu, WM164, WM793 were kindly provided by Professor Meenhard Herlyn from The Wistar Institute (Philadelphia, USA) and A2058, SKMEL5 and UACC62 were originally obtained from the American Type Culture Collection (ATCC, USA). Cell lines were cultured in high-glucose Dulbecco’s modified Eagle’s medium (DMEM) (Thermo Fisher Scientific) supplemented with 10% foetal bovine serum (FBS) (Thermo Fisher Scientific). The cultures were maintained at 37°C in a humidified atmosphere containing 5% (v/v) CO_2_ until 80% confluence. Cell lines were passaged every 72 hours as per ATCC guidelines. Sub-confluent monolayers were dissociated using 0.5% trypsin-EDTA in 1X phosphate buffered saline (PBS) solution (137 mM NaCl, 10 mM Phosphate, 2.7 mM KCl, pH of 7.4) (Thermo Fisher Scientific).

Flow cytometry was used to identify the different cell sizes of melanoma cell lines. Cell lines with greater than 80% viability, were washed twice with FACS buffer (0.1% bovine serum albumin, 100 mM EDTA, 10 mM HEPES, phosphate-buffered saline) and immediately acquired in a Gallios flow cytometer (Beckman Coulter). Cell sizes were inferred from the respective forward scatter values for each cell line in an analysis performed using the Kaluza Analysis Software (Beckman Coulter). Additionally, the cell size of A2058 and SKMEL5 melanoma cell lines, during 5 consecutive passages, was measured in a Vi-CELL XR cell counter (Beckman Coulter).

### Healthy volunteers and metastatic melanoma patients

Twenty melanoma patients with metastatic disease, at baseline, prior to clinical treatment with targeted inhibitors or immunotherapies, were included in the present study (Table 1). Healthy volunteers and melanoma patients signed consent forms approved by the Human Research Ethics Committees at Edith Cowan University (No. 11543), Sir Charles Gairdner Hospital (No. 2013-246) and South Metropolitan WA Health Service. For each patient, two BD Vacutainer K2 EDTA tubes (BD Biosciences) were collected and shipped to our research laboratory for sample processing within 24 hours post blood collection. The first five millilitres of blood were discarded to avoid epithelial contamination.

### Spiking of melanoma cell lines into blood of healthy controls

The melanoma cell lines with the greatest differential cell diameter, A2058 and SKMEL5, were used to determine the efficiency of the slanted spiral microfluidic device to isolate melanoma cells spiked into blood from healthy donors. Cells were stained with CellTracker^™^ Red CMTPX dye (Thermo Fisher Scientific), and 1, 5, 10, 50 or 100 live-stained melanoma cells were manually counted and spiked into 8 mL of blood. Experiments were performed in triplicate for each melanoma cell line.

### Recovery and purity of the slanted spiral microfluidic device

Before processing the spiked blood samples through the slanted device, blood samples were incubated with red blood cell (RBC) lysis buffer (140 mM NH_4_Cl, 17 mM Tris, pH 7.65) for 10 mins at room temperature with continuous gentle mixing. Lysed RBCs were subsequently removed after centrifugation at 300 g for 5 min and the nucleated cell fraction was washed twice with 20 mL of PBS before resuspension into 16 mL (0.5x concentration) of PBS supplemented with 0.5% bovine serum albumin (0.5% BSA/PBS). Nucleated cells from blood were processed through the slanted spiral microfluidic device as described by Warkiani et al. [24] (Supplementary Figure 1). Briefly, the slanted chips were washed with ethanol 70% and PBS before being primed with 0.5% BSA/PBS solution prior to sample processing. Melanoma cells were isolated by processing 16 mL of the nucleated cells in 0.5% BSA/PBS solution through the slanted spiral device at an input flow rate of 1700 μL min^−1^ controlled by a syringe pump (PHD 2000, Harvard Apparatus).

The slanted spiral device allows recovery from two outlets: one containing the recovered melanoma cells (CTC outlet) and the other containing the remaining white blood cells (waste outlet). To assess the recovery rate of this spiral device, the cellular fraction from the CTC outlet was transferred to a 48-well plate (Greiner Bio-One), and centrifuged at 500 g for 5 mins. Recovery rates were determined by counting the CellTracker^™^ red-labelled cells in an inverted fluorescent microscope (Eclipse Ti-E, Nikon^®^) and the NIS-Elements High Content Analysis software, version 4.2 (Nikon^®^). Purity of the slanted enrichment was determined by counting the total number of WBCs in the CTC fraction using a haemocytometer.

To increase the purity of the CTC fractions, we evaluated the recovery rates and WBC background after processing the sample twice through the slanted device (2X slanted). For this, 50 labelled cells derived from A2058 and SKMEL5 cell lines were spiked into 8 mL of blood from 4 healthy donors. The cellular fraction of the CTC outlet from the first round of slanted enrichment was resuspended in a total volume of 6 mL of 0.5% BSA/PBS solution and re-processed through the same washed and primed slanted device at the same flow rate. Cell Tracker^™^ red-labelled melanoma cells were counted as above and WBCs were counted in the nucleated fraction prior to enrichment, and in the ‘CTC’ fraction after 1^st^ and 2^nd^ rounds of enrichment.

### Isolation of CTCs from metastatic melanoma patients using the slanted spiral device

A total of 8 mL of blood were processed through the slanted microfluidic device. CTCs were enriched by processing the sample twice through the slanted microfluidic device (2X slanted). After enrichment, RNA was isolated using RNeasy Mini Kit (Qiagen) and stored at −80°C until further use.

### Detection of melanoma CTCs by flow cytometry

An aliquot of 8 mL of blood was processed by multimarker flow cytometry to confirm the presence of CTCs after microfluidic enrichment, as described by Gray et al. [10]. For this, blood samples were processed once through the slanted spiral device (1X slanted) and the enriched CTC fraction was then assessed for melanoma CTC subpopulations using our multimarker flow cytometry assay and the same gating strategies as previously described [10]. A total of 1 × 10^5^ WBCs from the waste outlet of the slanted device, were used as an isotype control for CTCs from the same patient sample, enriched via the device. The total and subpopulation-specific CTC counts were compared pre- and post-enrichment for every patient. For this, two additional aliquots of 8 mL of blood, one as an isotype control and the other as a pre-enrichment test sample, were collected and processed at the same time and from the same patient as the samples used for microfluidic enrichment.

### Gene expression assay to confirm CTC isolation

As previously described by Reid et al. [40], the expression of a panel of 5 melanoma-specific genes were assessed to confirm the presence of melanoma CTCs after slanted enrichment. Briefly, RNA (6 μL) isolated from cells from the enriched ‘CTC fraction’, for both spiked samples and patient blood, was reverse transcribed to cDNA using SuperScript^®^ VILO^™^ master mix (Thermo Fisher Scientific, US) in a 10 μL final volume reaction. Subsequently, *MLANA*, *TYR*, *MAGEA3*, *ABCB5* and *PAX3* genes were preamplified in a reaction containing 5 μL of input cDNA, 0.2X of a mixture of the TaqMan assays targeting these genes and the TaqMan® PreAmp Master Mix (Thermo Fisher Scientific). Expression of each of the 5 genes was then determined by qPCR of the preamplified cDNA using commercially designed TaqMan Gene Expression assays for each target gene. The 18S rRNA gene was used as an endogenous control. Cycling conditions and fluorescence detection of these PCR reactions were carried out in a ViiA^™^ 7 Real-Time PCR system (Thermo Fisher Scientific) [40]. Expression values were represented as the reciprocal Ct value for each target gene and plotted in a heatmap using the HeatMapViewer module (version 13.9; Broad Institute) at the GenePattern platform [57]. RNA isolated from cells in the CTC outlet after processing the blood of healthy individuals and from a sample spiked with 20 A2058 cells were included as negative and positive controls, respectively.

### Immunofluorescence staining

Immediately after the second round of slanted enrichment, cells within the ‘CTC-enriched fraction’ were fixed by 10 min incubation with 4% Paraformaldehyde (PFA) and subsequently cytospun (Cytospin^™^ 4, Thermo Fisher Scientific) onto a glass slide at 2000 rpm for 5 mins. Cells were again fixed with 4% PFA for 10 mins, then incubated in a blocking buffer containing 10% normal donkey serum (NDS), 3% bovine serum albumin (BSA) and 0.2% Triton X-100 (TX-100) in PBS for 15 mins. Cells were then stained in a 1 hour incubation with blocking buffer and a cocktail of the following antibodies: rabbit anti-gp100 (clone EPR4864, Abcam), rabbit anti-MLANA/MART1 (clone EP1422Y, Abcam), rabbit anti-S100 (clone EP1576Y, Abcam), mouse anti-MCSP conjugated to Alexa Fluor® 647 dye (clone 9.2.27, BD Pharmingen^™^) and mouse anti-CD45 conjugated to Phycoerythrin (PE) (clone HI30, BD Pharmingen^™^). Following incubation, cells were washed three times with 0.2% TX-100/PBS solution, and incubated with a donkey anti-rabbit IgG antibody conjugated to Alexa Fluor® 488 dye (Abcam) in blocking buffer. Finally, cells were mounted using ProLong® Gold Antifade Mountant with DAPI (4',6-diamidino-2-phenylindole) nuclear stain reagent (Thermo Fisher Scientific). Slides were stored at 4°C, visualised and scanned using the Eclipse Ti-E inverted fluorescent microscope (Nikon^®^). Stained cells were analysed using the NIS-Elements High Content Analysis software, version 4.2 (Nikon^®^) by two trained researchers. Background subtraction parameters for each marker, derived from immunostaining of A2058 melanoma cells spiked into WBC fractions, were stringently applied to all stained slides from patients’ CTC-enriched samples. After background subtraction, signal for each fluorescent dye was also adjusted to enhance the staining for each marker and parameters were applied to all stained slides. Only cells with confirmed positive staining, after background subtraction, for either MCSP, MEL or both and negative for the leukocytic marker CD45 were deemed as melanoma CTCs by consensus of multiple authors.

### Statistical analysis

A two-tailed independent t-test was used to compare the mean cell diameters of A2058 and SKMEL cells. One-way ANOVA analyses were used to find statistical differences in the recovery rates for A2058 and SKMEL5 cell lines between different spiked concentrations after 1X slanted enrichment. Similarly, one-way ANOVA was used to determine the efficiency of the slanted device to deplete WBCs after the second round of enrichment with this device processing blood samples from controls and patients. Statistical significance was reached with a P <0.05.

## SUPPLEMENTARY MATERIALS FIGURES


