# Supplementary material for: Isolation and detection of circulating tumour cells from metastatic melanoma patients using a slanted spiral microfluidic device
Source: Oncotarget. 2017 Jun 27;8(40):67355–68. doi: 10.18632/oncotarget.18641 (PMC5620178; doi:10.18632/oncotarget.18641)
Supplement: Supplementary file 1 [file oncotarget-08-67355-s001.pdf]

Isolation and detection of circulating tumour cells from metastatic melanoma patients using a slanted spiral microfluidic device

SUPPLEMENTARY MATERIALS

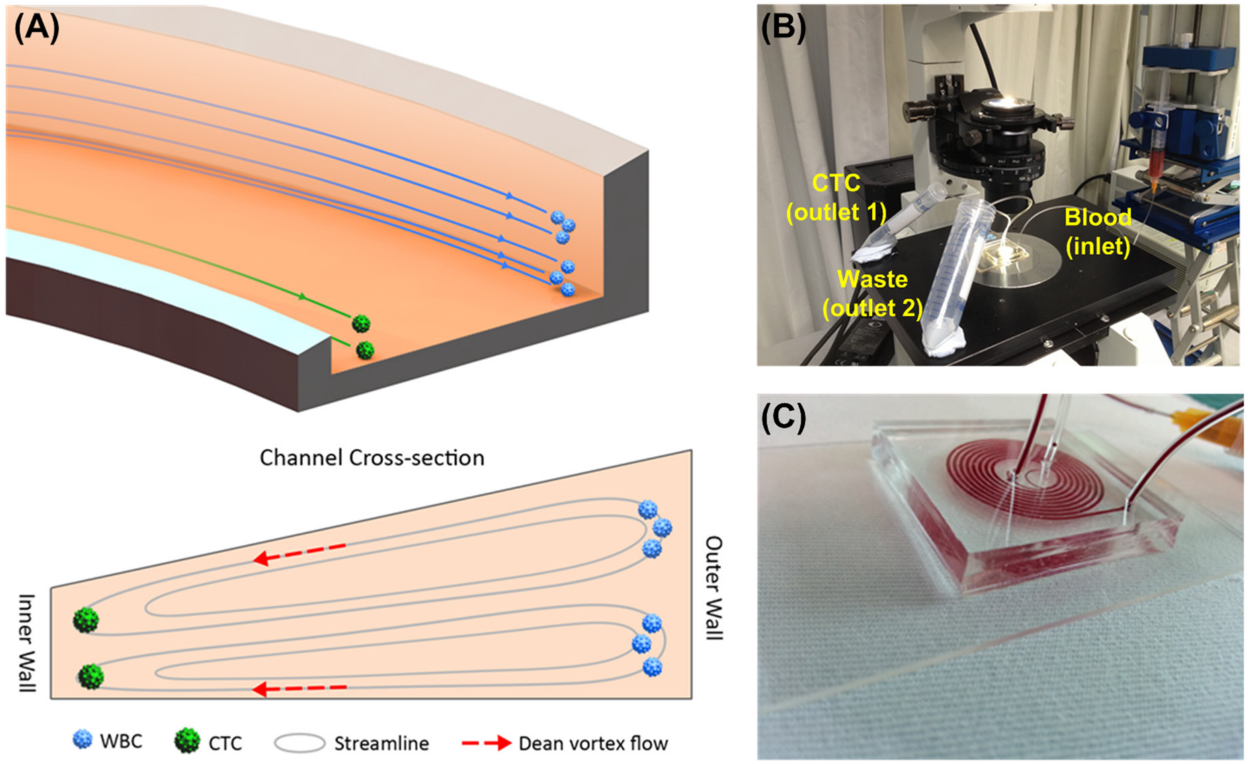

Supplementary Figure 1: (A) Schematic showing operation principle of CTC enrichment by a spiral channel with trapezoid cross-section. CTCs focused near the inner wall due to the combination of inertial lift forces and Dean drag forces at the outlet while leukocytes are trapped inside the core of dean vortex formed closer to the outer wall. **(B)** The workstation setup for CTC separation. The lysed blood is pumped through the spiral chip using a syringe pump where CTCs are separated from other blood components rapidly and efficiently. **(C)** Optical image of a spiral chip used in this work.

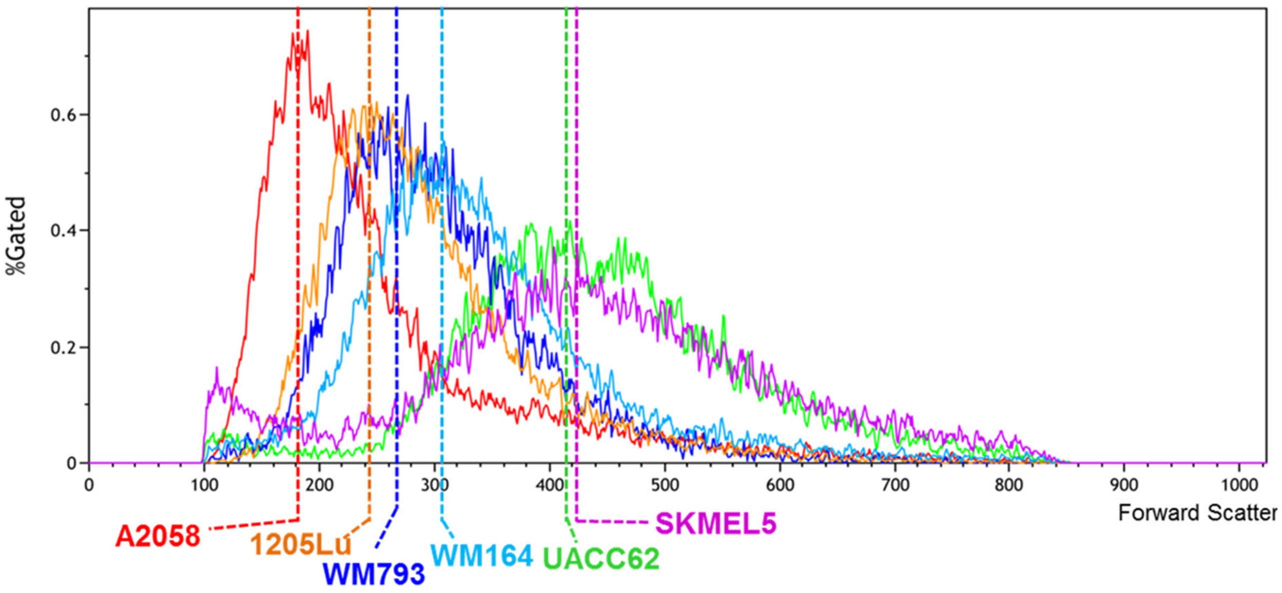

Supplementary Figure 2: Classification of 6 melanoma cell lines by size. Cell size for each melanoma cell line was inferred by forward scatter values using a flow cytometer.
